# Spatial, Temporal, and Functional Aspects of Macrophages during “The Good, the Bad, and the Ugly” Phases of Inflammation

**DOI:** 10.3389/fimmu.2014.00612

**Published:** 2014-12-03

**Authors:** Robert A. Harris

**Affiliations:** ^1^Applied Immunology and Immunotherapy, Department of Clinical Neurosciences, Karolinska Institutet, Karolinska Hospital, Stockholm, Sweden

**Keywords:** macrophage activation, M1/M2 macrophages, microenvironment, phenotype, immunotherapy, adoptive

In Sergio Leone’s classic western drama “*The Good, the Bad, and the Ugly*” the final scene depicts the three protagonists, each with their specific personality trait and fast-draw capability, assembled in a graveyard for a shoot-out. The analogy is thus to focal sites of inflammation, with different subpopulations of myeloid cells assembled within a tissue in proximity to each-other, but with different functional phenotypes, associated surface marker expression, and enacting different functions. The basic macrophage functional states are described as *pro-inflammatory*, *anti-inflammatory*, and *wound healing*, respectively. The M1 versus M2 phenotypic paradigm was first coined to distinguish macrophage populations and has been instrumental in increasing our knowledge of myeloid biology (1). Despite more recent suggestions that there is a continuum of activation states between the extremes of M1- and M2-type responses (2–4), this partly reflects an over-emphasis on cell surface phenotypes. We should now have the technologies to be able to assess the relevance of specific cells within specific microenvironments within a given healthy or diseased tissue. The issue of functionality (irrespective of surface phenotype) and the concept of functional diversity within distinct microenvironments within a tissue have been less studied, and is the focus of this commentary.

Let us first consider in simplistic terms three stages of an inflammatory response (Figure 1A): the “Good” non-inflammatory phase in which normal tissue homeostasis is maintained by resident macrophages (green arrows); the “Bad” phase in which potentially tissue-damaging macrophage functionality is initiated due to damage, infection, or autoimmunity (pro-inflammatory) or tumor development (anti-inflammatory) (red); and the “Ugly” phase representing a failure to down-regulate the initial response that results in chronic pathogenesis and tissue damage (blue), i.e., an inability to return to the “Good” phase through healing. The definition of “good” and “bad” in this sense will depend on the setting – a pro-inflammatory response to an infection may be desirable, but if uncontrolled may lead to tissue damage. Likewise, while anti-inflammatory responses beneficially modulate autoimmune reactions, they contribute to tumor development. The salient point is that macrophage function will vary during these different phases.

**Figure 1:**
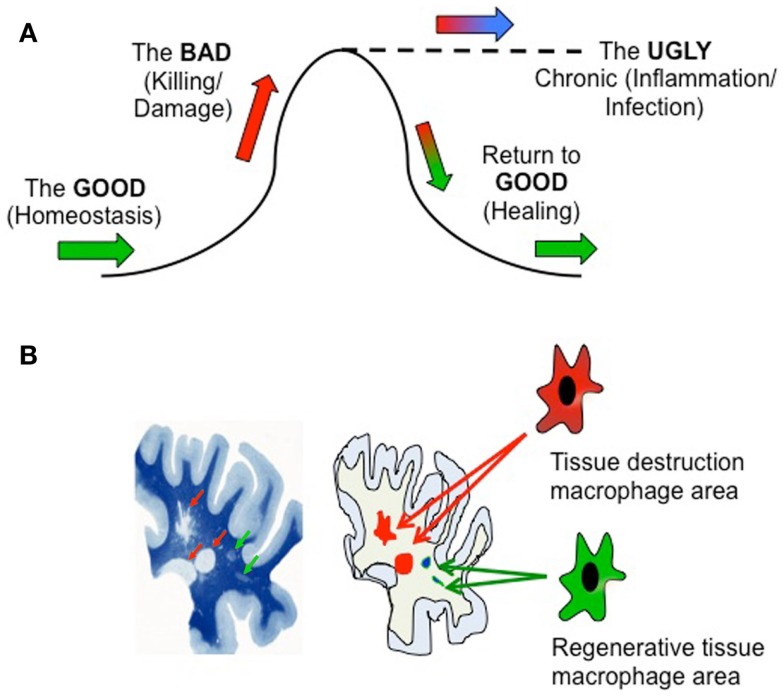
**(A)** Temporal and microenvironmental macrophage functionality during immune activation. **(B)** Left hand panel is reproduced, with permission, from Adams, C. W. M. A Colour Atlas of Multiple Sclerosis and Other myelin Disorders (©1989 Wolfe Medical Publications Ltd.).

## Microenvironmental Variation in Inflammation

The aforementioned basic view reflects the *overall* functionality of an inflamed tissue, but does not take into account the potential compartmentalization of a tissue, and that these processes may be occurring *simultaneously* in different areas of the affected tissue. Consider the image of a slice of multiple sclerosis subcortical white matter brain tissue (Figure 1B). Luxol fast blue staining (left) reveals healthy regions of myelin (dark blue), focal areas of demyelination (red arrows), and focal “shadow plaques” that represent remyelination (green arrows) (5). Two disparate processes – *pathogenic demyelination* and *healing remyelination*, occurring side-by-side, implicating that within these physically separate microenvironments immune cells such as macrophages might be conducting different processes – damage or repair (right image). In support of this, recent histopathological evidence reveals mixed macrophage phenotypes in human MS lesions (6).

Understanding of the concept of specific microenvironments within tissues is increasing, not least within tumor immunology (7). The ambiguous role of tumor associated macrophages (TAMs) in tumor progression is reflected by TAMs both actively augmenting cancer cell proliferation, invasion, metastasis, and angiogenesis by releasing cytokines, growth factors, enzymes, and angiogenic factors, but they also kill cancer cells. These varied activities encompass both M1 and M2 macrophage properties. It is counterintuitive that such diverse tumor-promoting, or conversely anti-tumoral, activities are performed by a single TAM cell type, so the existence of distinct TAM subpopulations associated with different intra-tumoral microenvironments is predicted (8). The source of the TAM may also influence their functions. For example, in gliomas TAM may be either resident brain microglia or blood infiltrating macrophages. Molecularly and functionally distinct TAM subpopulations may thus coexist in tumors, the heterogeneity depending on cancer type, stage of tumor progression and specific location within the tumor tissue (9, 10). A dynamic “switch” in TAM phenotype during tumor progression may explain the mixed activation state of TAM subsets present in different established tumors, and in certain models a switch from TAM is linked to tumor progression (11). It is noteworthy that the “switch” that is often referred to in the literature may rather reflect a *relative predominance* in M1 or M2 cell numbers rather than a full phenotypical/functional change of a single cell. Different macrophage populations induced during tumor progression have also been reported to occupy different microenvironments within the tumor mass (12). In a murine hepatocellular carcinoma model, the MHC Class II^high^ TAM population (M1-like) was associated with tumor growth suppression during early tumor growth while MHC II^low^ TAMs (M2-like) dominated as the tumor progressed (13).

Investigation of the spatial distribution of macrophage phenotypes in human plaques at different stages of atherosclerosis development also reveals microenvironment variations. M1 is the predominant phenotype in rupture-prone shoulder regions, and M2 in the adventitia (14). Likewise, in models of lung inflammation induced by butylated hydroxytoluene or *Mycobacterium tuberculosis* there is an initial M1 activation that progresses to M2 (15). However, granuloma-associated macrophages during active infection may retain an M1 phenotype while nearby uninfected alveolar macrophages are M2. Clearly, at various sites of inflammation then individual subpopulations of cells contribute to specific microenvironments in different ways.

## Inflammation is a Temporally Evolving Circumstance

Another aspect to consider when homogenizing tissues for cell purification and subsequent FACS or RT-PCR analyses is not only the geographical gradient of macrophage activation states that will be lost but also a temporal element. For example, given the realization that resident macrophages and circulating monocytes are fundamentally different cell types (16, 17), consider the macrophage disappearance reaction (18) in which initial immune activation within a tissue results in efflux of resident cells and infiltration of circulating cells, this cellular flux being reversed on eradication of the offending stimulant. If one would take a snapshot in the tissue and sample the phenotype of the macrophages present at a given time, it is difficult to be sure whether they are coming or going from the site of interest. Obviously, this can impact on their functional relevance to the ongoing immunological process and especially in our scientific interpretation. Now that small rodent PET/CT/MRI imaging is becoming more standardized, one can expect this issue to be further addressed in forthcoming years.

## Function Versus Form during Inflammation

A host requires basic macrophage functions in order to survive. These functions are sometimes less easily measured than other surrogate markers such as cell surface proteins. Consequently, there is a wide range of surface markers, cytokines and chemokines reported to distinguish M1 and M2 activation states (mostly *in vitro*). However, the use of such “markers” without parallel assessment of functions can result in conflicting results.

Variation in published results might be explained by variation in employed activation protocol, difference in rodent strain (19) or human donor to which the same protocol is applied. Take IL-10, for example, a prototypic anti-inflammatory cytokine that can be produced by M2 and M1 cells. Both the production and lack of production of IL-10 in M-CSF-stimulated human M2 monocytes have been described (21). Reliance on surface marker expression can also be particularly misleading – if anti-inflammatory M2 states receive an additional LPS stimulation *in vitro*, then while expression of CD86 and MHC II might become upregulated (“M1”) the IL-10 production is actually enhanced (22). Given the dominant functional role of IL-10 as an immunosuppressive cytokine, then functionally such cells are more potent M2, despite starting to develop an M1 surface. Similarly, in alcoholic hepatitis, liver M2 macrophages were determined to express M1-associated receptors (23). It would thus seem that there is a necessity to distinguish between surface and functional phenotypes.

Clearly, the biological functions in microenvironments should be more important than any other phenotype, and one expects as much functional variation as there is in M1–M2 phenotypic definition. Even the basic morphology of activated cells *in vitro* is reported to be exact for different phenotypes, yet closer examination reveals this is not necessarily the case (20, 24, 25). In our own study in which we applied IL-4, IL-10, and TGFβ simultaneously, cells of three different morphologies were apparent in the same culture well, representing the three distinct morphologies observed if single cytokines were applied in separate cultures (26). Does this imply microheterogeneity even in the cell culture medium containing a mixture of cytokines, such that the first cytokine receptor ligated on a cell surface dictates the morphology of that specific cell? Whether the sequence of activation is of any consequence will depend on whether there are actually any kinetic effects that impinge on the final morphology or function. This may seem a trivial issue but a recent publication elegantly demonstrated precisely why this is important by studying human macrophage transcriptomes using different combinations of activation stimulants (4).

The challenge is thus how to quantify functional phenotypes in microenvironments. We have a limited range of markers that can be applied immunohistologically. Detailed knowledge about expression/regulation of expression for *each macrophage population* in *each tissue*, within *each microenvironment* within a tissue, during *both* resting and inflammatory states, is currently lacking. Development of conditional knockout mouse strains lacking resident/peripheral macrophage populations is one approach that is warranted, for example, to distinguish the relative roles of infiltrating macrophages and resident microglia during brain tumor development. Alternatively, the use of single cell laser capture and proteomic or genetic analyses might be one modern approach to explore what individual cells within a particular microenvironment do within their niche, although function will only be inferred from these analyses (27, 28). The basic assumption is that all cells are equal and that the relative numbers of different subpopulations will ultimately define the functional state of the tissue. Increasing evidence challenges this assumption, and in our hands co-culture of pre-activated M1- and M2-type populations demonstrated a clear dominant phenotype of M2 cells (pre-activated with a combination of IL-4/IL-10/TGFβ) (29). Even if single cells are phenotyped and their relative numbers are quantified within a tissue or microenvironment, it remains difficult to predict the net functional activity or interplay *in vivo* if they are not functionally equivalent.

## Therapeutic Manipulation of Chronic Inflammatory Microenvironments

A final question is whether chronic inflammatory states such as autoimmune diseases represent a failure to down-regulate pro-inflammatory M1-mediated tissue destruction (i.e., a deficiency in anti-inflammatory M2 function), or whether this reflects a lack of healing M2 functionality. It follows that a stochastic alteration of the relative M1/M2 functions within microenvironments represents a feasible therapeutic approach. Earlier work indicated that M2 cells accumulate at the edge of the tissue damage in the setting of spinal cord injury (30). If large numbers of “therapeutic cells” could be applied to inflammatory microenvironments (e.g., through local stimulation or cell transfer) then they should be able to exert tailored “local” immunomodulatory effects. In our experience adoptively transferred pre-activated anti-inflammatory M2 cells resulted in clinical abrogation of both T1D (22) and MOG-EAE (26) disease courses. In addition to a cell therapy approach, there are new generations of agents aimed at specific conversion of macrophage phenotypes within microenvironments, such as TAM conversion to M1 within tumors using docetaxel and phosphatidylserine-targeting antibody (31). The effectiveness of this approach was first reported many years ago (32). The major challenge will be to access the microenvironments specifically rather than systemically administrating an agent and hoping for its specific access to the target area.

## Looking Back and Looking Ahead

The last decade has heralded a revolution in our understanding of immune mechanisms and particularly the critical role of macrophages in both innate and adaptive responses. During the coming decade, we can expect a refinement of this knowledge when the functions of individual cells and their specific contributions to a specific microenvironment become better understood. This may lead to yet further refinement of macrophage nomenclature as function supersedes form in importance. The next era may well also herald the successful therapeutic manipulation of inflammatory microenvironments in order to slow or abrogate inflammatory disease courses in human beings. One can be certain that the good macrophages vanquishing the bad macrophages will be a component aspect, and that restoration of the damaged, ugly tissue will also be macrophage-dependent.

## Conflict of Interest Statement

The author declares that the research was conducted in the absence of any commercial or financial relationships that could be construed as a potential conflict of interest.
